# Estimation of confidence intervals for decompositions and other complex demographic estimators

**DOI:** 10.4054/demres.2023.49.5

**Published:** 2023-07-11

**Authors:** Arun S. Hendi

**Affiliations:** 1 Princeton University, USA

## Abstract

**BACKGROUND:**

While the use of standard errors and confidence intervals is common in regression-based studies in the population sciences, it is far less common in studies using formal demographic measures and methods, including demographic decompositions.

**OBJECTIVE:**

This article describes and provides explicit instructions for using four different approaches for computing standard errors for complex demographic estimators.

**METHODS:**

Standard errors for Arriaga’s decomposition of life expectancy differences are computed using the delta method, the Poisson bootstrap, the binomial bootstrap, and the Monte Carlo approaches. The methods are demonstrated using a 50% sample of vital statistics data on age-specific mortality among urban women in the Pacific region of the United States in 1990 and 2019.

**RESULTS:**

All four methods for computing standard errors returned similar estimates, with the delta method, Poisson bootstrap, and Monte Carlo approaches being the most consistent. The Monte Carlo approach is recommended for general use, while the delta method is recommended for specific cases.

**CONTRIBUTION:**

This study documents multiple ways of estimating statistical uncertainty for complex demographic estimators and describes in detail how to apply these various methods to nearly any rate-based demographic measure. It also provides advice on when the use of standard errors is and is not appropriate in demographic studies. Explicit formulae for computing standard errors for Arriaga’s decomposition using the delta method approach are derived.

## 1. Introduction

Though statistical inference is commonly used in demographic studies involving regression analysis and some types of forecasting, it is far less likely to be applied to life table analyses, including those based on mortality, fertility, or any other types of rates; decompositions across age, duration, cause of death, proximate determinants, or other indices; and studies involving more complex formal demographic measures.

The avoidance of statistical inference in some demographic studies is likely due to four separate reasons. First, it is in part due to the fact that demography as a field predates modern statistical inference (and certainly the widespread use of statistical hypothesis testing), meaning that many common demographic methods were developed without attention to uncertainty arising from sampling variance. Second, demographers often employ data that theoretically capture 100% of the population (e.g., national vital statistics records), meaning there should be zero sampling variance. Third, the intricate mathematics and theoretical assumptions underlying some demographic models often make it difficult to derive closed-form estimators for sample variance or even to identify precisely what the random variable in an analysis should be. Finally, unlike statistics, demography is a substantive field, where the norm is to make judgments based on expertise. Well-trained demographers know when a life expectancy is reasonable or whether the difference between two countries in the total fertility rate is meaningful. They thus focus more on demographic significance than on statistical significance. Demographers are typically more concerned with problems arising from measurement error, which in this line of work often affects estimates far more than sampling variance ever could.

With the increasing level of interdisciplinarity in population research and publishing, demographers are more often being called on to provide standard errors, which can sometimes be a challenging task. No major demography textbook provides advice on precisely how to compute standard errors for complex demographic estimators, and the statistical literature is often either impenetrable or poorly adapted for demographic studies. Several past studies have provided helpful descriptions of procedures to compute standard errors for standardized rates, life expectancies, and multistate life table parameters (Andreev and Shkolnikov 2010; Chiang 1984; Keyfitz 1966; Lo, Dan Vatnik, and Bourbeau 2016). This study adds to this literature by deriving an explicit equation for approximating the variance of Arriaga’s (1984) decomposition using the delta method; detailing the assumptions and methods for computing empirical standard errors for any demographic estimator using Monte Carlo simulations and tools such as the parametric bootstrap and the jackknife; and comparing and contrasting variance estimates based on different approaches. While this article uses Arriaga’s decomposition as an example of how to apply various variance estimation methods, the approaches described in this paper can be applied to any estimator that uses demographic rates or probabilities as its input.

The remaining sections of this article describe when standard errors are required; how to use the Monte Carlo, bootstrap, delta method, and jackknife approaches to computing standard errors; how these approaches compare; and how the various approaches can be extended to compute standard errors for any probability or rate-based demographic estimator. Readers who are simply interested in getting a precise description of how to compute Monte Carlo standard errors for demographic estimators can skip directly to subsection 3.2, and those who are looking for a closed-form expression for approximate standard errors for Arriaga’s decomposition can proceed to the subsection labeled 3.1.

## 2. When are standard errors necessary?

Standard errors are important tools for assessing the precision of a sample-based estimator. They allow researchers and their readers to understand whether an effect, trend, or difference arises from underlying population processes versus sampling variation. When standard errors or other measures of uncertainty are not provided in sample-based studies, the researcher leaves open the possibility that a result or conclusion is indistinguishable from a lack of result or a different conclusion.

When using whole population data, however, standard errors are generally not necessary (see discussions of finite sample inference in Lohr 2022; Abadie et al. 2020, and elsewhere). For example, if a researcher is using data on the total population and all births in the year 2009 in California to compute the state’s 2009 crude birth rate, there is no need to compute standard errors since there is no sampling variation (because there is no sample). The purpose of standard errors is to capture variation that could occur due to random sampling, so it is the random sampling, and not the underlying random data-generating process, that drives the variation of interest in the estimator. An estimate based on a 100% sample of the target population has a standard error of zero.

In some cases, demographers may work with whole population data pertaining to small areas or relatively small countries. Either the researcher or a reader may want to know more about the degree of random variation of an estimate. In such a case, the population parameter is fixed, so the researcher might wish to avoid computing a standard error for the value. In these instances, the researcher can instead answer a related question that would arrive at the same conclusion: If my population were instead to be a random sample of the same size being drawn from an infinitely large population, what would the standard error be for my estimate? In that case, one can use a method like Monte Carlo simulation, described in the subsequent pages, to provide an answer. Producing standard errors to answer this type of hypothetical question has precedent in demography, going at least as far back as Keyfitz (1966).

More generally, standard errors are but one paradigm through which a researcher can judge the quality of an estimate or the difference between two estimates. When applied without thought, the use of standard errors can be misleading. For example, it would not be sensible to compare an estimate based on data known to be faulty with an estimate based on nonfaulty data, even if the “standard errors” were very large or very small (Hendi 2017). Demographers have their own, separate set of standards to judge the quality of measurement, and in general those should take precedence over concerns about sampling variance.

As general guidance, standard errors (or confidence intervals or other measures of sampling variance) should be used when employing sample data to make inferences about population parameters or when making comparisons of estimates from two or more samples. Standard errors can be interpreted as indicators of the precision of an estimate in the sense that an estimate with a smaller standard error can be thought of as more likely to be representative of the underlying population parameter.

## 3. Approaches for computing standard errors

We focus on four main approaches to constructing standard errors or confidence intervals for decompositions and other complex demographic estimators: the delta method approach, Monte Carlo simulation, two versions of the parametric bootstrap, and the jackknife. These are the most commonly used methods for approximating sample variance in social science research, but they see relatively less use in demography. We describe each method in turn and demonstrate how to apply several of the methods to compute standard errors for Arriaga’s age decomposition of the difference between two life expectancies. We then compare the approaches, describing the strengths and weaknesses and indicating the appropriate use case for each approach.

### 3.1 The delta method approach

The delta method is an analytic approach to approximating the sample variance of an estimator. We say it is analytic because it has a closed-form expression and doesn’t depend on resampling, unlike many empirical standard error approaches. The logic behind the delta method is to compute the sample variance of a linearized version of an estimator, since deriving a formula for the variance of a linear function of estimators is often easier than computing the variance of a nonlinear function (Greene 2018). Consider population parameter θ (e.g., the survival probability at ages 45–49). We use sample data to estimate that parameter, and our estimator is $\overset{ˆ}{\theta }$, which is asymptotically normal. We are ultimately interested in some continuous function of the parameter, g(θ) (e.g., life expectancy at birth), and thus would like to compute the sample variance of $g(\overset{ˆ}{\theta })$. Rather than undertake that complicated computation, we instead approximate the estimator by writing:

$$g(\hat{\theta })\approx g(\theta )+(\hat{\theta }-\theta )\cdot g^{\prime }(\theta )$$

and write the variance of this approximation as:

$$var(g(\hat{\theta }))\approx var(\hat{\theta })\cdot {\left(g^{\prime }(\theta )\right)}^{2}.$$

To compute the approximate sample variance of a function of some estimator, we multiply the variance of the estimator itself by the square of the derivative of the function. This logic can be extended to functions of multiple estimators, as in the case of many demographic measures that are functions of multiple age-specific rates or probabilities.

To apply this approach to Arriaga’s decomposition, we first write the decomposition as a function of the age-specific estimators for survival or mortality (the ${\;}_{n}p_{x}$ or ${\;}_{n}m_{x}$ values).^2^ These age-specific estimators are asymptotically normal. We can write Arriaga’s decomposition in terms of ${}_{n}p_{i}$ values as follows:

$${\;}_{n}{\text{Δ}}_{x}={\;}_{n}p_{0}^{1}\times \ldots \times {\;}_{n}p_{x-n}^{1}\cdot \left({\;}_{n}a_{x}^{2}+{\left(n-{\;}_{n}a_{x}^{2}\right)}_{n}p_{x}^{2}\right)-{\;}_{n}p_{0}^{1}\times \ldots \times {\;}_{n}p_{x-n}^{1}\;\cdot \left({\;}_{n}a_{x}^{1}+{\left(n-{\;}_{n}a_{x}^{1}\right)}_{n}p_{x}^{1}\right)+e_{x+n}^{2}\times {\;}_{n}p_{x}^{2}\cdot \left({\;}_{n}p_{0}^{1}\times \ldots \times {\;}_{n}p_{x-n}^{1}\right)\;-e_{x+n}^{2}\times {\;}_{n}p_{0}^{1}\times \ldots \times {\;}_{n}p_{x-n}^{1}\times {\;}_{n}p_{x}^{1}$$

to allow for straightforward differentiation with respect to the ${\;}_{n}p_{i}^{1}$ and ${\;}_{n}p_{i}^{2}$ values (where the superscripts correspond to the two populations whose life expectancy difference is being decomposed). The ${\;}_{n}a_{x}$ value represents the average number of person-years lived by someone who dies in the age interval x to x+n. Following the above notation, the g function is ${\;}_{n}\Delta_{x}$ and the θ values are the ${\;}_{n}p_{i}^{1}$ and ${\;}_{n}p_{i}^{2}$ values that we are differentiating with respect to. Those derivatives are used to calculate the variance. The ${\;}_{n}p_{i}^{1}$ and ${\;}_{n}p_{i}^{2}$ values appear in this formulation of the decomposition in two ways: either through the ${\;}_{n}p_{i}$ values themselves or implicitly through the $e_{x+n}$ values. We thus additionally note that the derivative of $e_{x+n}$ is

$$\frac{\partial }{\partial_{n}p_{i}}e_{x+n}=\{\begin{array}{cc} 0 & \text{if}\;x+n>i\\ \frac{l_{i}}{l_{x+n}}\cdot \left[\left(n-{\;}_{n}a_{i}\right)+e_{x+2n}\right] & \text{if}\;x+n\leq i. \end{array}$$

Then the derivatives of ${\;}_{n}\Delta_{x}$ with respect to the parameter values are:

$$\frac{\partial_{n}{\text{Δ}}_{x}}{\partial_{n}p_{i}^{1}}=\{\begin{array}{cc} \frac{l_{x}^{1}}{l_{0}^{1}}\cdot \frac{1}{{\;}_{n}p_{i}^{1}}\cdot \left(\frac{{\;}_{n}L_{x}^{2}}{l_{x}^{2}}-\frac{{\;}_{n}L_{x}^{1}}{l_{x}^{1}}\right)+\frac{e_{x+n}^{2}}{{\;}_{n}p_{i}^{1}}\cdot \left({\;}_{n}p_{x}^{2}\cdot \frac{l_{x}^{1}}{l_{0}^{1}}-\frac{l_{x+n}^{1}}{l_{0}^{1}}\right) & \text{if}\;x>i\\ -\frac{l_{x}^{1}}{l_{0}^{1}}\cdot \left(\left(n-{\;}_{n}a_{x}^{1}\right)+e_{x+n}^{2}\right) & \text{if}\;x=i\\ 0 & \text{if}\;x<i \end{array}$$

$$\frac{\partial_{n}{\text{Δ}}_{x}}{\partial_{n}p_{i}^{2}}=\{\begin{array}{cc} \frac{l_{x}^{1}}{l_{0}^{1}}\cdot \left(\left(n-{\;}_{n}a_{x}^{2}\right)+e_{x+n}^{2}\right) & \text{if}\;x=i\\ \frac{l_{i}^{2}}{l_{x+n}^{2}}\cdot \left(\left(n-{\;}_{n}a_{i}^{2}\right)+e_{x+2n}^{2}\right)\times \left({\;}_{n}p_{x}^{2}\cdot \frac{l_{x}^{1}}{l_{0}^{1}}-\frac{l_{x+n}^{1}}{l_{0}^{1}}\right) & \text{if}\;x<i\\ 0 & \text{if}\;x>i. \end{array}$$

The open-ended age group has to be treated specially. Since ${\;}_{\infty }q_{x}=1$, some prior studies assumed zero variance arising from this age group (Chiang 1984). In many instances, the open-ended age group encompasses a significant proportion of all deaths, suggesting that it does contribute variance to demographic measures (Lo, Dan Vatnik, and Bourbeau 2016). We thus instead assume that deaths in this age group arise from a Poisson process in which the variance of the number of deaths equals the mean. The derivatives involving mortality in the open-ended age group are thus:

$$\frac{\partial_{n}{\text{Δ}}_{x}}{\partial_{\infty }D_{\omega }^{1}}=0\;\;\;\;\;\;\;\text{if }x<\omega$$

$$\frac{\partial_{n}{\text{Δ}}_{x}}{\partial_{\infty }D_{\omega }^{2}}=-\frac{l_{\omega }^{2}}{l_{x+n}^{2}}\cdot \frac{e_{\omega }^{2}}{{\;}_{\infty }D_{\omega }^{2}}\cdot \left({\;}_{n}p_{x}^{2}\cdot \frac{l_{x}^{1}}{l_{0}^{1}}-\frac{l_{x+n}^{1}}{l_{0}^{1}}\right)\;\;\;\text{if}\;x<\omega$$

$$\frac{\partial_{\infty }{\text{Δ}}_{\omega }}{\partial_{n}p_{i}^{1}}=\frac{l_{\omega }^{1}}{l_{0}^{1}}\cdot \frac{1}{{\;}_{n}p_{i}^{1}}\cdot \left(e_{\omega }^{2}-e_{\omega }^{1}\right)\;\;\text{if}\;i<\omega$$

$$\frac{\partial_{\infty }{\text{Δ}}_{\omega }}{\partial_{n}p_{i}^{2}}=0\;\;\;\;\;\;\text{if}\;i<\omega$$

$$\frac{\partial_{\infty }{\text{Δ}}_{\omega }}{\partial_{\infty }D_{\omega }^{1}}=\frac{l_{\omega }^{1}}{l_{0}^{1}}\cdot \frac{e_{\omega }^{1}}{{\;}_{\infty }D_{\omega }^{1}}$$

$$\frac{\partial_{\infty }{\text{Δ}}_{\omega }}{\partial_{\infty }D_{\omega }^{2}}=-\frac{l_{\omega }^{1}}{l_{0}^{1}}\cdot \frac{e_{\omega }^{2}}{{\;}_{\infty }D_{\omega }^{2}}$$

The variance of the Arriaga’s decomposition estimator can thus be approximated using the delta method by

(1) $$\text{var}\left({\;}_{n}{\text{Δ}}_{x}\right)\approx \left[\sum_{i=0}^{\omega -n}{\left(\frac{\partial_{n}{\text{Δ}}_{x}}{\partial_{n}p_{i}^{1}}\right)}^{2}\times \left(\frac{{\left(1-{\;}_{n}p_{i}^{1}\right)}^{2}\times {\;}_{n}p_{i}^{1}}{{\;}_{n}D_{i}^{1}}\right)\right]\;+\left[\sum_{i=0}^{\omega -n}{\left(\frac{\partial_{n}{\text{Δ}}_{x}}{\partial_{n}p_{i}^{2}}\right)}^{2}\times \left(\frac{{\left(1-{\;}_{n}p_{i}^{2}\right)}^{2}\times {\;}_{n}p_{i}^{2}}{{\;}_{n}D_{i}^{2}}\right)\right]\;+{\left(\frac{\partial_{n}{\text{Δ}}_{x}}{\partial_{\infty }D_{\omega }^{2}}\right)}^{2}\times {\;}_{\infty }D_{\omega }^{2}$$

(2) $$\text{var}\left({\;}_{\infty }{\text{Δ}}_{\omega }\right)\approx \left[\sum_{i=0}^{\omega -n}{\left(\frac{\partial_{\infty }{\text{Δ}}_{\omega }}{\partial_{n}p_{i}^{1}}\right)}^{2}\times \left(\frac{{\left(1-{\;}_{n}p_{i}^{1}\right)}^{2}\times {\;}_{n}p_{i}^{1}}{{\;}_{n}D_{i}^{1}}\right)\right]\;+\left[{\left(\frac{\partial_{\infty }{\text{Δ}}_{\omega }}{\partial_{\infty }D_{\omega }^{1}}\right)}^{2}\times {\;}_{\infty }D_{\omega }^{1}\right]+\left[{\left(\frac{\partial_{\infty }{\text{Δ}}_{\omega }}{\partial_{\infty }D_{\omega }^{2}}\right)}^{2}\times {\;}_{\infty }D_{\omega }^{2}\right].$$

In practice, applying the delta method to Arriaga’s decomposition, or any demographic estimator involving an age-structured population, becomes somewhat complicated in that it requires keeping track of two indices: the age index of the estimator, x, and the age index of each parameter, *i*. For Arriaga’s decomposition applied to standard abridged life tables, this means that for each of the ${\;}_{n}\Delta_{x}$ values for the 19 age groups we must calculate derivatives corresponding to each of the 19 ${\;}_{n}p_{x}$ and ${\;}_{\infty }D_{\omega }$ parameters for each of the two populations involved in the decomposition. Clearly, using the delta method for demographic measures can quickly become complicated.

How then, in practice, do we apply the delta method to compute the sample variance for Arriaga’s decomposition? First we compute the decomposition itself. Then, for each ${\;}_{n}\Delta_{x}$ value, we use the above formulas to calculate the derivatives with respect to each age group, evaluated at the observed life table values. We then plug those values into Equations (1) and (2) above. We provide an Excel spreadsheet with formulas as well as R code, each demonstrating these computations for two abridged life tables, in the supplementary online material. Researchers can replace the life table parameters in the worksheet or data files with their own data to retrieve standard errors corresponding to their analyses.

The delta method procedure can easily be adapted for cases when the researcher is using multistage stratified sample surveys instead of simple random samples. The only changes in these cases would be that in Equations (1) and (2) one would replace $\left(\frac{{\left(1-{\;}_{n}p_{i}^{1}\right)}^{2}\times {\;}_{n}p_{i}^{1}}{{\;}_{n}D_{i}^{1}}\right)$ and $\left(\frac{{\left(1-{\;}_{n}p_{i}^{2}\right)}^{2}\times np_{i}^{2}}{{\;}_{n}D_{i}^{2}}\right)$ with the respective squared standard errors of the ${\;}_{n}p_{i}^{1}$ and ${\;}_{n}p_{i}^{2}$ values, where the standard errors for these values are supplied by the statistical software. Also in Equations (1) and (2), one would replace ${\;}_{\infty }D_{\omega }^{1}$ and ${\;}_{\infty }D_{\omega }^{2}$ with the respective squared standard errors of ${\;}_{\infty }D_{\omega }^{1}$ and ${\;}_{\infty }D_{\omega ;}^{2}$; once again these standard errors are supplied by the statistical software.^3^ So long as the researcher has made sure to set up the rate or probability estimation analysis in the software to take into account survey design and sample weights (e.g., in Stata this would be by using the **svyset** command and **svy** prefixes, and in R one would use the survey package), this method will return accurate standard error estimates for Arriaga’s decomposition.

### 3.2 The Monte Carlo approach

Another approach to approximating the sample variance of a demographic estimator is through Monte Carlo simulation. Monte Carlo methods work by simulating estimates based on distributional assumptions. They are often used to assess the properties of sampling distributions for complex estimators that may not admit closed-form standard errors (Hendry 1995). In the context of demography, Monte Carlo approaches have been used to study the sampling distributions of life table parameters (Andreev and Shkolnikov 2010).

For a complex demographic estimator such as Arriaga’s decomposition, we can apply a Monte Carlo approach by making an assumption about the sampling distribution of ${\;}_{n}p_{x}$ or ${\;}_{n}m_{x}$ values, drawing a random value from those distributions for each age group, computing Arriaga’s decomposition based on each set of age-specific random draws, recording the decomposition values, and then repeating the process hundreds or even thousands of times. The hundreds or thousands of distinct decomposition values make up the sampling distribution of the decomposition estimator under the distributional assumptions mentioned earlier. For example, the standard deviation of this empirical sampling distribution is the standard error for the decomposition. The 2.5^th^ and 97.5^th^ percentiles of the empirical sampling distribution correspond to the 95% confidence interval values.

Applying the Monte Carlo approach is a straightforward three-step procedure. The first step is to determine the parameters and their corresponding estimators. For Arriaga’s decomposition (and just about any other demographic estimator), the parameters could be either the ${\;}_{n}p_{x}$ values for all age groups or the ${\;}_{n}m_{x}$ values for all age groups. Since most life tables are constructed starting from ${\;}_{n}m_{x}$ values, we use those as our running example. If one is using, for example, a 1% sample of the population to estimate ${\;}_{n}m_{x}$ values, then one can assume that age-specific deaths follow a Poisson distribution, where the mean and variance are both ${\;}_{n}D_{x}$ (the observed number of deaths in the sample). The age-specific population counts, ${\;}_{n}N_{x}$, are assumed to be nonrandom.

The second step is to draw a random number of deaths $\left({\;}_{n}D_{x}\right)$ for each age group and for each of the two populations involved in the decomposition from the respective Poisson distribution for that group. Next we compute the life tables for the two populations based on the simulated data, and then we compute Arriaga’s decomposition based on these two simulated life tables. We then record the decomposition values and repeat step two 999 more times, recording the decomposition values each time. If we were to conduct 1,000 simulations, then at the end we should have recorded 1,000 ${\;}_{n}\Delta_{x}$ values for each age group, giving us an empirical sampling distribution for each age group.

The third and final step is to compute the value of interest based on the simulated estimates. For example, if we wanted to know the standard error for the decomposition value at ages 50–54, we would calculate the standard deviation of the simulated decomposition values for ages 50–54. If we wanted the 95% confidence interval of the decomposition estimate for the 50–54 age group, we would compute the 2.5^th^ and 97.5^th^ quantiles of the simulated decomposition values for ages 50–54.

Though we have outlined the Monte Carlo approach using a Poisson distribution assumption for death rates, the Poisson assumption is likely to hold just as well for any type of demographic rate, including fertility, marriage, migration, or other types of transitions. In some cases, however, it may be more appropriate or easier to use alternate distributional assumptions. For example, if we were to compute age-specific death rates using event history analysis (either occurrence-exposure ratios or model-based estimates) or some other type of maximum likelihood estimation, it might be sensible to instead assume a normal distribution for the rate estimators themselves.

One remaining question is how to choose the number of simulations. We mentioned above that one could compute hundreds or even thousands of simulations in the Monte Carlo exercise. There are many rules of thumb, but 1,000 simulations should be adequate for most demographic estimators. The runtime for computing Monte Carlo standard errors for Arriaga’s decomposition based on 1,000 simulations should be no more than a few seconds on a modern computer. We provide sample R code to compute Monte Carlo standard errors in the supplementary online appendix.

The description above has emphasized the use of Monte Carlo for simple random samples, but Monte Carlo approaches are of equal utility for researchers using multistage stratified sample survey data. In the context of demographic estimation, the most common way this type of data is used is by producing rate or probability estimates using the survey data and subsequently using these rate or probability estimates as inputs to compute decompositions, life expectancies, or other quantities, often stratified by group. For example, one could use data from the U.S. National Health Interview Survey to estimate age-specific death rates by sex, education category, and five-year period and then use these rate estimates to compute an age decomposition of the change over time in the educational gradient in life expectancy. This is a very complicated estimator, and most statistical packages would provide only the sample variances of the rates themselves, not those for the decomposition. Furthermore, the researcher may not be sure which decompositions they want to compute until after running their code to estimate the rates and then examining those rates. They may also want to compute follow-up estimates at a later stage.

In these cases, the Monte Carlo approach is the most flexible and easy-to-use method for computing standard errors. Most statistical packages offer options to incorporate survey design and sample weights when dealing with complex survey data. Thus, when one uses survey functions to compute means, ratios, probabilities, or predicted values from regressions, the statistical software will return not just the estimates but also standard errors that take into account sample design. The rate or probability estimates from these analyses are asymptotically normally distributed, with mean equal to the estimate itself and sample variance equal to the squared standard errors. When the researcher subsequently wishes to compute a decomposition or other quantity that involves the rates or probabilities previously estimated, they can estimate standard errors for the decomposition by employing the Monte Carlo approach: They would simply draw random rates or probabilities from normal distributions, with means and variances provided by the rate or probability estimates and their squared standard errors, and then compute the decomposition based on these randomly drawn values. Repeating this process 999 additional times and computing the standard deviation of the 1,000 recorded values yields the standard error of the decomposition. The value of this approach is that it does not require researchers to rerun an entire analysis every time they wish to compute a new decomposition (which, when dealing with large event history data files, can be very computationally intensive). Instead the rates or probabilities and their corresponding standard errors have to be estimated only once. The Monte Carlo standard error for the decomposition or another complex demographic estimator will thus take into account survey design, so long as the estimator is entirely a function of quantities estimated in the first stage of analysis (e.g., rates or probabilities). If the complex demographic estimator is a function of additional estimates, then variance arising from those estimates should also be incorporated into the Monte Carlo exercise.

### 3.3 The bootstrap approach

The bootstrap is a commonly used approach to estimating sample variance that relies on resampling. It was first introduced by Efron (1979) as an alternative to the popular jackknife procedure. There are two common types of bootstrap procedures: the nonparametric and the parametric bootstrap. The logic behind the nonparametric bootstrap is that one can replicate the sampling process by taking a with-replacement sample of size *N* from any population-representative sample of size *N*. Because sample variance represents the variation in an estimator when applied across different potential samples, the nonparametric bootstrap approach mirrors the sampling process when the original sample size (*N*) is large enough. In the parametric bootstrap procedure, the researcher makes a distributional assumption about the data-generating process to replicate the sampling process. Another way to word this is that one assumes a model from which the data arise and then uses the estimated parameters of the model to create new synthetic samples, repeating the process to generate a sampling distribution. The sample size in each synthetic sample is equal to the sample size of the original sample. Both the parametric and nonparametric approaches can be used to produce confidence intervals for complex demographic estimators.

To apply the nonparametric bootstrap, one needs to replicate the sampling process. For example, when working with a 1% simple random sample of annual vital statistics data, the researcher would first organize the data so that each row represents an individual, including an indicator for whether or not the individual died in the period of interest. The data are then sorted into age groups. If there are *N* rows in the data for a given age group, researchers would then randomly sample *N* of the rows with replacement, so that any given row can be selected multiple times. The same procedure would be applied to each age group, producing a replicate sample that contains observations for each age group. For each replicate sample, the researcher would compute the decomposition or other estimator of interest, record the value, and then repeat the procedure with a new replicate sample.

Applying the nonparametric bootstrap to complex survey data is a bit more complicated, since each survey has its own distinct design, meaning that two different surveys could potentially require two very different bootstrap resampling procedures. Since many surveys today provide instructions for drawing bootstrap samples, one can easily apply those procedures to produce a replicate sample. For one specific way to construct bootstrap weights for a multistage complex probability sample, see Lohr (2022:376–77). One would then produce a split records (person-year) file for the replicate sample and carry out the decomposition or other estimation, record the result, and start again with a new replicate sample. The set of results from each replicate sample constitutes the sampling distribution. One can select the 2.5^th^ and 97.5^th^ percentiles of this distribution to form a 95% confidence interval.

The parametric bootstrap requires less in the way of sampling, since it assumes a specific data-generating process. If a researcher is using a 1% sample from vital statistics data, for example, they can apply the parametric bootstrap by assuming that age-specific deaths are generated from a Poisson process with the intensity parameter equal to the number of deaths in the sample for that age group. The researcher would then draw a random number of deaths for each age group from the corresponding Poisson distribution. This latter step is the parametric analog to drawing a with-replacement sample with size equal to the original sample size. The researcher would next compute age-specific death rates based on these randomly generated death counts and 1% of the age-specific population count (the latter is assumed to be nonrandom), compute the decomposition or other demographic estimator of interest, record the result, and then start again with a new set of random draws from the age-specific Poisson distributions, repeating this hundreds or thousands of times. Once again, these results constitute the sampling distribution, and one can select the 2.5^th^ and 97.5^th^ percentiles of this distribution to form a 95% confidence interval. We do not give instruction on applying the parametric bootstrap to complex survey data, since doing so is more onerous and provides no clear advantage over the nonparametric bootstrap.

The Monte Carlo and bootstrap approaches are very similar, and in fact the bootstrap can be thought of as a special case of the Monte Carlo approach. The main difference is that the bootstrap requires knowledge of the data or the data-generating process, while one can get by using the Monte Carlo approach based only on knowledge of the sampling distributions of the age-specific rate or probability estimators. For example, suppose a researcher was interested in computing a decomposition of the difference between the life expectancies for college graduates in France and the United Kingdom. The researcher finds estimates and standard errors for the age-specific rates in a published paper but doesn’t have access to the original survey data used to produce the estimates. That researcher can instead assume that the estimators are approximately normal^4^ and draw random rate estimates from a normal distribution with mean equal to the reported rate and variance equal to the square of the reported standard error. They could apply the Monte Carlo approach using this assumption, which would allow them to compute confidence intervals for their decompositions without any reference to the original survey data. In addition to requiring less (or no) data, this method is also easier to apply with complex survey data than the nonparametric bootstrap, which requires an intimate understanding of the sampling procedure and can take hours or even days to run on a sufficiently large sample with thousands of bootstrap replicates.

### 3.4 The jackknife approach

Another approach commonly used to produce uncertainty estimates is the jackknife. Like the nonparametric bootstrap, the jackknife relies on replicating the estimator using multiple subsamples of the data (see section 11.5.5 of Cameron and Trivedi 2005 for an accessible description). When using a simple random sample of size *N*, the “delete-one” jackknife is a replication approach where the researcher excludes one observation (the $j^{\text{th }}$ observation) at a time, recomputes the estimator using this subsample, records the estimate (denoted ${\;}_{n}\Delta_{x}(j)$), and then repeats this procedure (N-1) more times, excluding the next observation each time. The jackknife estimate of sample variance is then $\frac{N-1}{N}$ multiplied by the sum of the squared differences between each jackknife replicate estimate and the overall estimate:

$$\text{var}\left({\;}_{n}{\text{Δ}}_{x}\right)\approx \frac{N-1}{N}\sum_{j=1}^{N}{\left({\;}_{n}{\text{Δ}}_{x}(j)-{\;}_{n}{\text{Δ}}_{x}\right)}^{2}.$$

When using data from a complex multistage stratified sample, the approach differs slightly, since this type of sample design requires the researcher to keep all observations together within each primary sampling unit (PSU). Thus, instead of excluding one observation at a time, the researcher excludes one PSU at a time, inflating the sample weights to account for the exclusion of the PSU. Different surveys have varying recommendations for how to apply the jackknife when using their data. See Lohr (2022:374) for one clear description of how to apply the jackknife to sample survey data generally.

The jackknife is a useful tool for estimating sample variance primarily because of its simplicity of application. For the purposes of decompositions and other complex demographic estimators applied to large simple random samples of vital statistics data, it is less useful. For example, applying the jackknife to a sample of 5,000,000 would require estimating the decomposition 5,000,000 times, which could take thousands of times longer than the approaches described above. The jackknife is more useful when estimating variance for complex estimators applied to sample surveys with relatively small numbers of PSUs, such as the Demographic and Health Surveys (Elkasabi 2019; Lohr 2022). In the case of non-smooth estimators, such as sample quantiles, the delete-one jackknife may not be consistent or asymptotically unbiased (Shao and Wu 1989), rendering the application of the jackknife much more difficult. Because the jackknife does not present a good use case for large vital statistics samples compared to the methods described above, it is not included in the numerical comparison that follows, but it should be considered a useful option for complex survey data.

## 4. Comparison of approaches and application to demographic decomposition

Researchers may wonder: Which of these variance estimation methods is best for my specific application? While this section goes through the steps of demonstrating each method and comparing the resultant standard errors, the reader may be happy to hear that all the approaches described in this article return very similar standard error estimates, meaning that demographers can choose any one of the methods with confidence that it will not substantially affect the findings or interpretation of results. All the methods also allow for the incorporation of complex survey design when stratified multistage sample data are used, so that the standard error estimates can reflect the sampling approach. We can thus focus on other dimensions when comparing the variance estimation methods, including ease of use, concordance with other methods, accuracy, computational efficiency, and consistency across multiple applications.

This section compares and contrasts the various sample variance estimation approaches described above using example data on female mortality in the urban portion of the Pacific region of the United States. The data consist of a 50% simple random sample of age-specific death and population counts from the years 1990 and 2019. The counts are sorted into the age groups 0, 1–4, 5–9, 10–14, …, 80–84, and 85+ years. Deaths data come from the annual U.S. Multiple Cause of Death (MCD) files, and population counts come from the annual bridged-race population estimates. The goal of the analysis is to compute Arriaga’s decomposition for the change in life expectancy at birth between those two years for women in this region. Between 1990 and 2019, life expectancy increased by 5.7 years for women in this region. Table 1 presents the decomposition of this 5.7-year increase into age group contributions, with most of the increase attributable to declines in infant mortality and mortality at ages 50 and older.

One may wish to establish the precision of these estimates, either out of curiosity or because of a request from a reader. We can do this using any of the three approaches described above. We provide R code or computations in Excel for each approach in the supplementary online appendix. We start with the Monte Carlo approach.

For our Monte Carlo standard errors, we draw random age-specific death rate values and compute Arriaga’s decomposition for each set of age-specific draws. We assume that the underlying deaths in this population arise from a Poisson distribution.^5^ Under this assumption, both the mean number of deaths and the variance in the number of deaths will equal the observed number of deaths. Rather than draw random deaths directly from a Poisson distribution, we use the fact that the Poisson distribution is well approximated by a normal distribution. Since the age-specific death rate is computed as ${\;}_{n}m_{x}={\;}_{n}D_{x}/{\;}_{n}N_{x}$ (where ${\;}_{n}D_{x}$ and ${\;}_{n}N_{x}$ are the death and population counts, respectively, from the 50% sample – that is, not life table or stationary-equivalent values), then the death rate will have an approximate normal distribution with mean equal to ${\;}_{n}m_{x}$ and variance equal to $\frac{{\;}_{n}D_{x}}{{\;}_{n}N_{x}^{2}}={\;}_{n}m_{x}/{\;}_{n}N_{x}$. For each age group, we draw 1,000 replicate ${\;}_{n}m_{x}$ values from the age-specific normal distribution with mean ${\;}_{n}m_{x}$ and variance ${\;}_{n}m_{x}/{\;}_{n}N_{x}$ for the 1990 sample and then do the same for the 2019 sample. We then use each set of mortality schedules to compute 1,000 different 1990 life tables and 1,000 different 2019 life tables. Finally, we compute 1,000 Arriaga’s decompositions for the difference between the corresponding 1990 and 2019 life tables, recording the decomposition values as we go. For each age group, we compute the standard deviation of the $1,000{\;}_{n}\Delta_{x}$ values, which is our estimate of the standard error for the Arriaga’s decomposition value at ages x to x+n. The standard error for the sum of the ${\;}_{n}\Delta_{x}$ values (the standard error for the change in life expectancy between 1990 and 2019) can be computed by summing up the ${\;}_{n}\Delta_{x}$ values for each of the 1,000 decompositions and computing the standard deviation of these 1,000 values.

The second approach we demonstrate is the delta method approach. Applying this method is very straightforward, since it only requires the researcher to plug life table values into the equations given above. The challenging aspect of applying the delta method to Arriaga’s decomposition is that one must keep track of two separate indices: the age index for the decomposition value (x to x+n) and the age index for the derivative (i to i+n). In this application, we assume that deaths are binomially distributed in each age group from infancy through 80–84 years and that deaths at ages 85+ are Poisson distributed. We compute derivatives of Arriaga’s decomposition with respect to the ${\;}_{n}p_{i}$ and ${\;}_{\infty }D_{85}$ values for each of the two populations (1990 and 2019) and the variances for the ${\;}_{n}p_{i}$ and ${\;}_{\infty }D_{85}$ values for the same two populations before combining them in the summations shown in Equations (1) and (2). When applying the method in our example case, using a spreadsheet for computation, we use an intermediate step to compute $\frac{l_{i}^{2}}{l_{x+n}^{2}}\cdot \;\left(\left(n-{\;}_{n}a_{i}^{2}\right)+e_{x+2n}^{2}\right)$, which appears in the derivative with respect to ${\;}_{n}p_{i}^{2}$. The intermediate step is not required but makes incorporating $e_{x+2n}^{2}$ simpler in a spreadsheet format. The intermediate step uses an alternate way to construct a life expectancy and is related to an approach used by Chiang (1984). The computations for the delta method application are provided in an Excel file in the supplementary appendix; sample R code is in a separate file.

The third approach for approximating the variance of Arriaga’s decomposition is the bootstrap, and we apply two different versions of the parametric bootstrap. The first version assumes that deaths are distributed binomially while the second assumes that deaths arise from a Poisson distribution. For each of the 1,000 or so replicate samples in the binomial version, we draw random numbers of age-specific deaths from age-specific binomial distributions with probability ${\;}_{n}q_{x}$ and with the number of trials equal to ${\;}_{n}D_{x}/{\;}_{n}q_{x}$ for all but the open-ended age group. (Since ${\;}_{\infty }q_{85}=1$, the variance in the open-ended age group under the binomial assumption would be zero.) For the open-ended age group, we draw a random number of deaths from the Poisson distribution with parameter ${\;}_{\infty }D_{85}$. We use this random draw strategy for each of the two periods, 1990 and 2019. For each replicate sample, we construct death rates and life tables and then compute Arriaga’s decomposition and record the values. The Poisson version is very similar. The only difference is that in the Poisson version, the random death counts at ages 0 through 80–84 are drawn from the Poisson distribution with parameter ${\;}_{n}D_{x}$. The resulting decomposition values constitute the sampling distribution of the Arriaga’s decomposition estimator, and the standard deviation of those values equals the standard error.

Table 1 shows the results of applying Arriaga’s decomposition to the change in life expectancy between 1990 and 2019 based on the 50% sample data from each of those years. For a particular age group, Arriaga’s decomposition provides the number of years of the life expectancy difference attributable to changes in mortality for that age group. The values are highest for infancy and at the older adult ages and are relatively low for ages 1 through 50. The sum of the decomposition values across all age groups is 5.66 years, which is equal to the overall change in life expectancy between 1990 and 2019.

**Figure 1:**
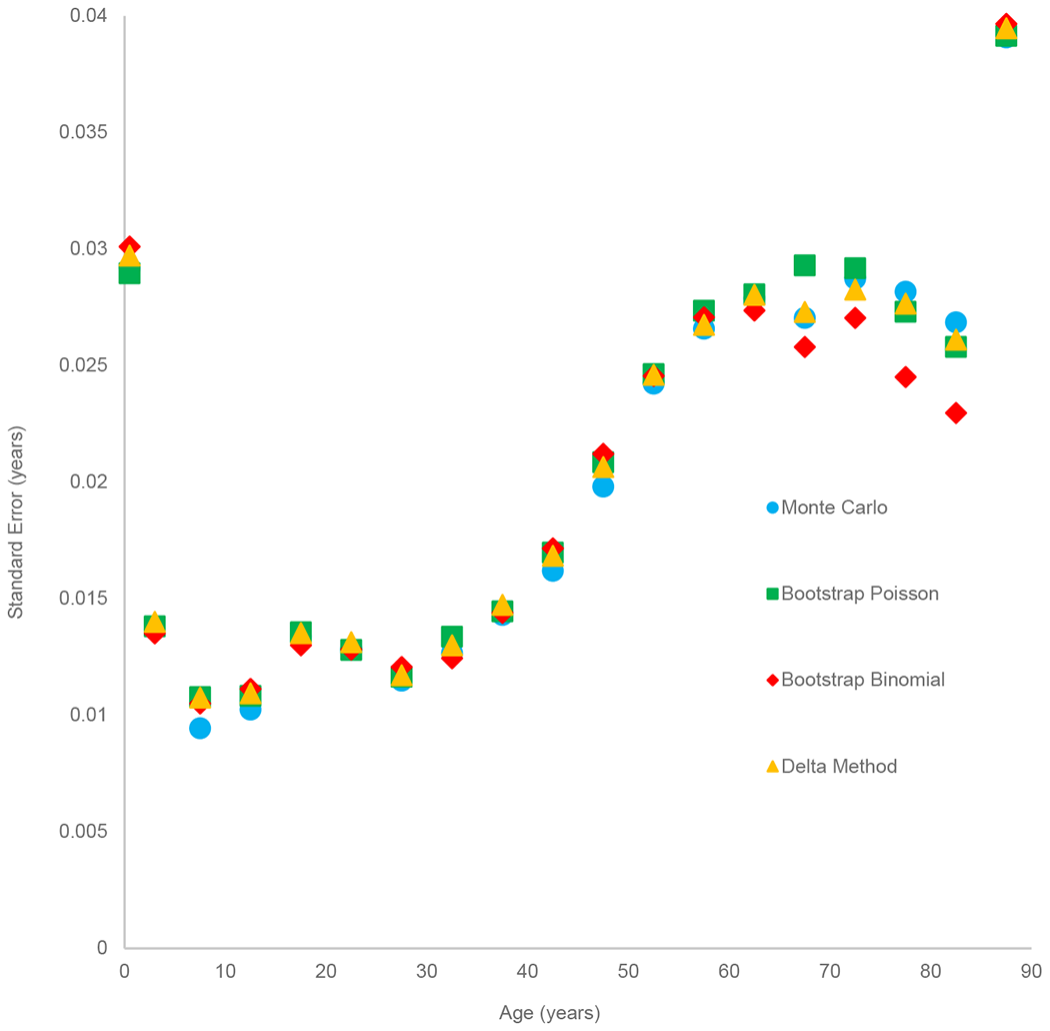
Comparison of standard errors for Arriaga’s decomposition using different statistical approaches *Note*: Monte Carlo assumes that age-specific death rates follow a normal distribution, with mean equal to the observed death rate and variance equal to $\frac{{\;}_{n}m_{x}}{{\;}_{n}N_{x}}$. Bootstrap binomial and delta method assume that deaths are binomially distributed for age groups 0 through 80–84 and that Poisson is distributed for ages 85+.

Figure 1 plots standard error estimates for Arriaga’s decomposition as a function of age for each of the four methods described above. The age pattern of the standard error estimates tends to follow the age pattern of the Arriaga decomposition values – the largest standard errors are at infancy and at the older adult ages while smaller standard errors prevail at the child and younger adult ages. The older-age deceleration in standard errors starting at around age 60 is in part due to the greater number of deaths occurring at those ages relative to the preceding age groups. A larger number of deaths is akin to a larger sample size, leading to lower sample variance. From infancy through ages 60–64 and for ages 85+, the standard error estimates are nearly identical for all four types of estimators. At ages 65–69 through 80–84, the bootstrap method using the binomial assumption tends to produce standard errors that are slightly lower than estimates produced using the other three methods, which all produce highly similar standard error estimates. Theoretically, the binomial bootstrap should return standard errors that are comparable to the delta method approach, since both approaches use the same distributional assumptions. The lower standard error produced by the binomial bootstrap may be due to that method’s different approach to randomly drawing the number of deaths in a synthetic cohort, which relies on equating the number of trials to ${\;}_{n}D_{x}/{\;}_{n}q_{x}$. The other methods do not require specifying the number of trials in this way.

**Figure 2:**
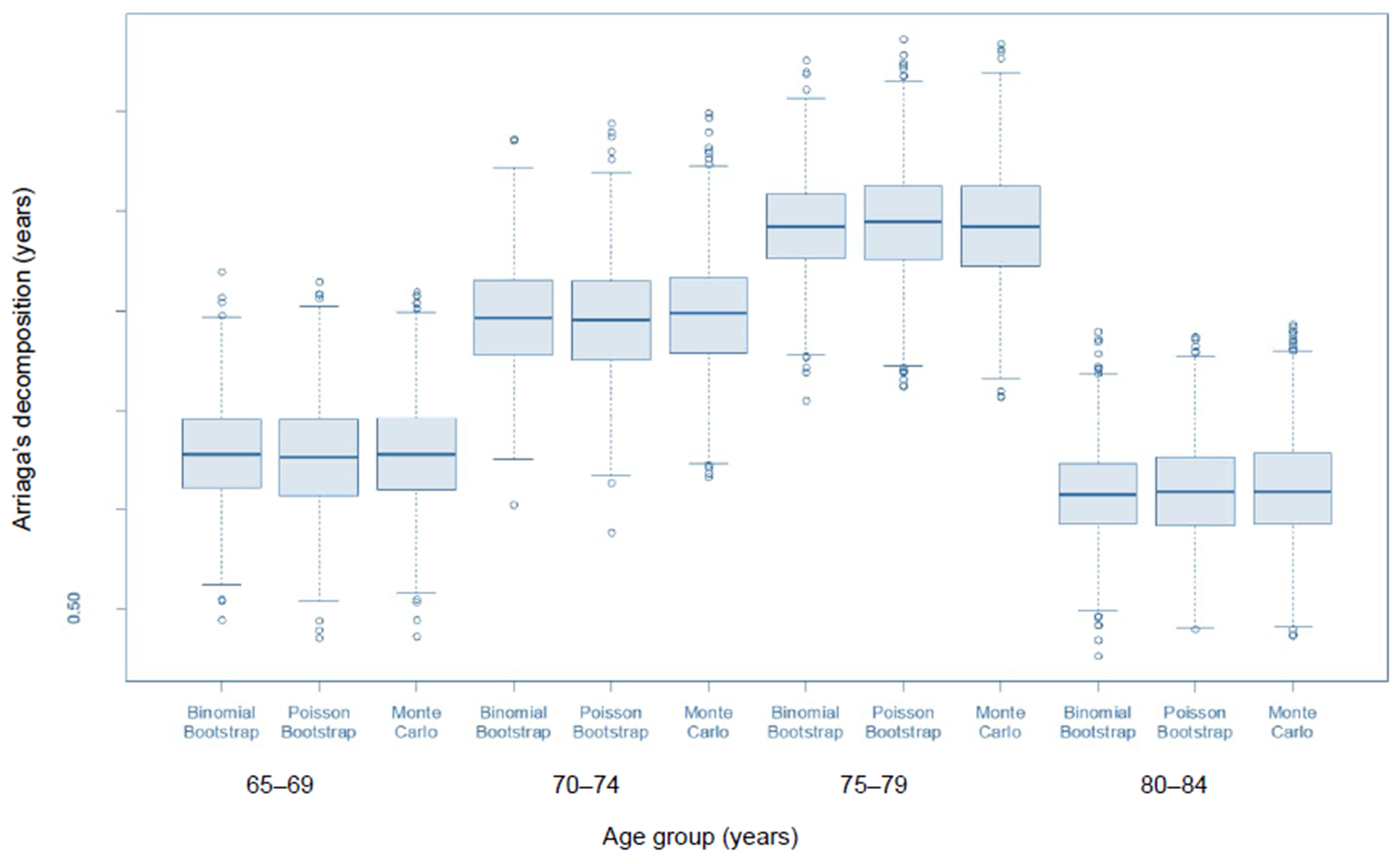
Empirical sampling distributions of Arriaga’s decomposition from bootstrap and Monte Carlo simulations, ages 65–69 through 80–84

Figure 2 shows boxplot diagrams for each of the empirical standard error methods (Monte Carlo and bootstrap) in the 65–69 through 80–84 age groups. Each point used to compute the boxplot is an Arriaga decomposition value from one of the 1,000 simulations, so the boxplots represent the empirical sampling distributions of the decomposition estimator. It is clear from this figure that the centers of the distributions are all highly comparable; the spreads of the Poisson bootstrap and Monte Carlo sampling distributions are also highly similar. The spread of the binomial bootstrap is somewhat lower, which is reflected in the lower standard error described above. The lower variance is not being driven solely by outliers in the extremes of the distribution but by a smaller interquartile range as well.

Despite the small differences between the four methods, and in particular between the binomial bootstrap and the other three approaches, all four methods provide very similar standard error estimates, with the Monte Carlo, Poisson bootstrap, and delta method approaches being the most consistent.

At the end of it all, the reader may again ask: Which of these approaches is best for me to use? We can adjudicate the different methods on the basis of six dimensions: accuracy, data requirements, concordance with other methods, ease of use, computational efficiency, and consistency across multiple applications. Table 2 summarizes the comparisons across five of these dimensions. The easiest of these dimensions to judge is accuracy. All four methods are accurate in the sense that they all provide theoretically correct estimates, although it should be noted that all four methods are approximations. A complex estimator like Arriaga’s decomposition does not admit an exact closed-form sample variance formula. Another important limitation is that none of these four methods assigns positive variance when zero deaths (or births, etc.) are observed. In these cases, the estimated death rate will be zero and the sample variance will either be zero or undefined. Though all four methods are theoretically accurate, we saw that the Poisson bootstrap, delta method, and Monte Carlo approaches tended to be in concordance more often, suggesting that these methods should be preferred over the binomial bootstrap.

Of the four methods, the Monte Carlo approach is perhaps the easiest to use. It requires assumptions that are similar to those of the other methods and is very easy to code, leaving less room for potential mistakes. The delta method approach is perhaps the most difficult to use, since it requires the researcher to compute derivatives of potentially very complex estimators. The counterargument to ease of use is computational efficiency. The delta method is by far the most computationally efficient of the approaches described above. Because it doesn’t require simulation or repeated sampling, it will have the quickest runtime. The remaining methods do not differ greatly from one another in their computational efficiency. Finally, the delta method is also the best approach in terms of consistency across multiple applications. Since it does not rely on empirical sampling, the delta method will always yield the same standard error when applied to the same estimator and data. The remaining three methods, on the other hand, will in general produce slightly different standard errors depending on where the random number generator in the statistical software is set.

Since all the approaches provide similar standard error estimates, perhaps a prudent tactic is to go with the simplest approach that provides the least room for error or confusion. We recommend using the Monte Carlo approach for three main reasons. First, the Monte Carlo approach requires very little in the way of complicated computations and makes only a very minimal additional assumption compared to the other methods. The delta method assumes that the death rate or death probability estimators are approximately normal, which is an excellent assumption. The Monte Carlo and bootstrap methods both make assumptions about the distribution for the data-generating process (that is, a binomial or Poisson distribution). The specific Monte Carlo approach outlined in this section additionally assumes that the age-specific death rates are approximately normal, which is in general a very good assumption so long as the number of deaths is around five or more.

The second advantage to the Monte Carlo approach is that it can be consistently applied across multiple types of demographic data. One can assume a normal distribution and apply the Monte Carlo approach to vital statistics samples, as shown above, or to estimates from an event history analysis (survival analysis) applied to survey data. Applying the delta method to event history analysis estimates requires replacing the sample variance terms for ${\;}_{n}p_{x}$ and ${\;}_{\infty }m_{85}$ in Equations (1) and (2) with estimated sample variances provided by the statistical software used to conduct the event history analysis. Applying the nonparametric bootstrap approach to event history analysis can become very complicated when one uses highly complex surveys, since the sampling methodology would need to be empirically replicated in the bootstrap analysis.

The third advantage of the Monte Carlo approach follows from the first two: One can compute Monte Carlo standard errors for estimates even without access to the underlying data used to produce those estimates. So long as the researcher has the rate (or probability) estimates and the standard errors for those rates (probabilities), they can use the Monte Carlo approach to construct confidence intervals even for estimators that are very complex functions of the rates or probabilities, such as Arriaga’s decomposition. While researchers typically do have access to the underlying data, the real benefit here arises from the way demographers typically conduct their analyses. They often start by estimating age- and category-specific rates or probabilities. At a later stage, they use those rates to compute a decomposition or other measure. In the first stage of this analysis, the researcher has already estimated the rates and the standard errors for those rates. For any subsequent calculations that require these rates as inputs, the researcher can simply use the rate standard errors in combination with Monte Carlo to produce the standard error for subsequent estimates of decompositions, etc. They do not need to run the entire analysis again. This is especially helpful when one is using event history analysis applied to large survey datasets to compute rates, which can be very computationally intensive. We thus recommend using the Monte Carlo approach whenever the researcher has a sufficient number of deaths (greater than five per age group) in the sample. When there are fewer than five deaths in some of the age groups, none of the methods described in this article (or anywhere else) will yield sensible standard errors, so the researcher should pick a method and explain that readers should take the estimates with a grain of salt, since the number of deaths is relatively low in a particular age group. The one exception to this advice is if one is writing a general purpose program to compute standard errors that will potentially be applied many times for a given estimator. For example, if one were to program an R package that computes a specific decomposition, it might be more sensible to simply hardcode the delta method approach into the package rather than require users to run a bootstrap or Monte Carlo analysis every time they use the decomposition function. (See the supplementary materials for sample R code for precisely this purpose.)

## 5. Extensions to other demographic estimators

The methods described above can be used for more general applications, including ascertaining standard errors for other very complex demographic estimators. The simplest extension is for approximating the standard error for Arriaga’s decomposition applied to causes of death. A cause of death decomposition uses age-cause-specific mortality rates. Arriaga’s cause of death decomposition is

$${\;}_{n}{\text{Δ}}_{x}^{i}={\;}_{n}{\text{Δ}}_{x}\cdot \frac{{\;}_{n}m_{x}^{i}(2019)-{\;}_{n}m_{x}^{i}(1990)}{{\;}_{n}m_{x}(2019)-{\;}_{n}m_{x}(1990),}$$

where i indicates cause of death i, so that ${\;}_{n}m_{x}^{i}={\;}_{n}D_{x}^{i}/{\;}_{n}N_{x}$ and the ${\;}_{n}\Delta_{x}^{i}$ values sum across i to ${\;}_{n}\Delta_{x}$. We can use the Monte Carlo approach to compute standard errors for the cause of death decomposition. One can assume that age-cause-specific deaths $\left({\;}_{n}D_{x}^{i}\right)$ are Poisson distributed and proceed just as described above by randomly drawing age-cause-specific death rates and computing the corresponding cause decomposition 1,000 times. The analysis would draw 1,000 random ${\;}_{n}m_{x}^{i}$ values from a normal distribution with mean ${\;}_{n}m_{x}^{i}$ and variance ${\;}_{n}m_{x}^{i}/{\;}_{n}N_{x}$ for each age-cause group and then compute the cause of death decomposition for each of the 1,000 sets of age-cause-specific rates. The standard deviation of the 1,000 simulated ${\;}_{n}\Delta_{x}^{i}$ values is the standard error for the cause of death decomposition value for cause i at ages x to x+n.

The general algorithm for computing Monte Carlo standard errors for any rate-based demographic estimator is given below:

1. Assume that the events process (age/cause/duration-specific deaths, births, marriages, migrations, divorces, etc.) follows a Poisson distribution.
2. Compute the event rates (death rates, birth rates, etc.).
3. Simulate new sets of age- or duration-specific event rates by drawing random rates from a normal distribution with mean equal to the observed rate and variance equal to the observed rate divided by the number of person-years of exposure.^6^ If the rates are estimated from complex survey data, let the variance equal the squared standard error of the estimated rate provided by the statistical software.
4. For each set of simulated duration- or age-specific rates, compute the estimator of interest (e.g., a decomposition, an age-standardized rate, a tempo-adjusted value, the number of person-years lost). Repeat steps three and four 1,000 times.
5. Record each simulated value of the estimator. The 2.5^th^ percentile and 97.5^th^ percentile of these values represent the lower and upper bounds of the 95% confidence interval for the estimator. The standard deviation of the simulated values is the standard error.

## 6. Conclusions

Standard errors or other estimates of statistical uncertainty are important components of demographic analyses when one is using sample data. This article describes four distinct methods for computing standard errors for complex, rate-based demographic estimators while also warning that standard errors can at times be inappropriate or misleading when used in demographic studies. We suggest that researchers use the Monte Carlo approach described above to quantify uncertainty when necessary.

## Supplementary Material

Supplementary Material

## Figures and Tables

**Table 1:** Arriaga’s decomposition for change in life expectancy between 1990 and 2019 for women in the urban Pacific region of the United States

	1990					2019					Arriaga
Age *x*	${\;}_{n}m_{x}$	${\;}_{n}a_{x}$	${\;}_{n}q_{x}$	*l_x_*	${\;}_{n}L_{x}$	${\;}_{n}m_{x}$	${\;}_{n}a_{x}$	${\;}_{n}q_{x}$	*l_x_*	${\;}_{n}L_{x}$	${\;}_{n}{\text{Δ}}_{x}$
0	0.007655	0.074435	0.007601	1.000000	0.992964	0.003609	0.063104	0.003596	1.000000	0.996631	0.34
1	0.000433	1.510379	0.001730	0.992399	3.965320	0.000175	1.516522	0.000698	0.996404	3.983887	0.08
5	0.000185	2.500000	0.000926	0.990682	4.951116	0.000118	2.500000	0.000588	0.995708	4.977077	0.03
10	0.000224	2.698390	0.001119	0.989765	4.946274	0.000109	2.673004	0.000546	0.995123	4.974350	0.04
15	0.000399	2.625958	0.001994	0.988657	4.938605	0.000208	2.717355	0.001042	0.994580	4.970533	0.06
20	0.000467	2.560885	0.002330	0.986685	4.927819	0.000327	2.599822	0.001635	0.993543	4.963819	0.04
25	0.000538	2.609242	0.002686	0.984386	4.915609	0.000366	2.632594	0.001829	0.991919	4.955300	0.05
30	0.000752	2.645253	0.003754	0.981742	4.900032	0.000562	2.652385	0.002807	0.990105	4.944000	0.05
35	0.001069	2.651170	0.005331	0.978056	4.878036	0.000781	2.646976	0.003897	0.987326	4.927576	0.07
40	0.001541	2.706326	0.007677	0.972843	4.847083	0.001120	2.649646	0.005585	0.983479	4.904482	0.09
45	0.002628	2.706583	0.013063	0.965374	4.797949	0.001600	2.669022	0.007968	0.977985	4.871762	0.19
50	0.004234	2.670296	0.020964	0.952764	4.717286	0.002449	2.688760	0.012179	0.970193	4.823654	0.29
55	0.006295	2.681929	0.031022	0.932790	4.596873	0.003895	2.677877	0.019302	0.958377	4.748930	0.33
60	0.010252	2.655346	0.050056	0.903853	4.413187	0.005957	2.648850	0.029376	0.939879	4.634479	0.48
65	0.015119	2.644060	0.072993	0.858610	4.145398	0.008548	2.662720	0.041901	0.912269	4.472003	0.58
70	0.023492	2.640862	0.111291	0.795938	3.770714	0.013564	2.673357	0.065747	0.874044	4.236520	0.65
75	0.037778	2.614471	0.173272	0.707357	3.244402	0.022154	2.685871	0.105370	0.816579	3.883780	0.69
80	0.061530	2.500000	0.266634	0.584792	2.534146	0.040466	2.500000	0.183743	0.730536	3.317103	0.56
85	0.151531		1.000000	0.428867	2.830227	0.110591		1.000000	0.596305	5.391965	1.05

Total					79.31					84.98	5.66

**Table 2:** Comparison of methods for computing standard errors of demographic estimators

A. Simple Random Sample of Vital Statistics Data					
Method	Data requirements	Computation time	Ease of use	Accuracy	Consistency across multiple applications
Delta Method	Rates + standard errors and deaths^a^	Single computation	Requires user to do some calculus	Accurate	Exact
Monte Carlo	Rates and their standard errors	1000 × decomposition runtime	Easy	Accurate	Consistent but not exact
Jackknife	Original sample	Proportional to sample size	Mostly easy	Accurate	Exact
Poisson Bootstrap	Original sample	1000 × rate estimation and decomposition runtime	Mostly easy	Accurate	Consistent but not exact
Binomial Bootstrap	Original sample	1000 × rate estimation and decomposition runtime	Mostly easy	Mostly accurate	Consistent but not exact
B. Multistage Stratified Sample of Survey Data					
Method	Data requirements	Computation time	Ease of use	Accuracy	Consistency across multiple applications
Delta Method	Rates + standard errors and deaths^a^	Single computation	Requires user to do some calculus	Accurate	Exact
Monte Carlo	Rates and their standard errors	1000 × decomposition runtime	Easy	Accurate	Consistent but not exact
Jackknife	Original sample	Proportional to # PSUs	Mostly easy	Accurate	Exact
Poisson Bootstrap	Original sample	1000 × rate estimation and decomposition runtime	Mostly easy	Accurate	Consistent but not exact
Binomial Bootstrap	Original sample	1000 × rate estimation and decomposition runtime	Mostly easy	Mostly accurate	Consistent but not exact

a This assumes that the derivative of the estimator is a function of rates and deaths, as in the case of Arriaga’s decomposition.

*Note*: Cells highlighted in green indicate that a method is the best performer on the column metric, while blue highlighting indicates that the method is a close second best. The multistage sample bootstrap methods being compared are the nonparametric versions.

## Footnotes

2 The notation used in this article is similar to that used in Preston, Heuveline, and Guillot (2001).

3 Note that one should change only values that show up directly in Equations (1) and (2). The ${\;}_{\infty }D_{\omega }$ values in the partial derivatives should not be changed.

4 This will in general be a good assumption if the ${\;}_{n}p_{x}$ values aren’t too close to 0 or 1 and if the number of agespecific person-years in the sample is sufficiently large. One rule of thumb suggests that the normal approximation to the binomial distribution is accurate if np and n(1-p) are both greater than 5. In the case of survey-based mortality estimates, this means we should observe at least five deaths and five survivors in any given age group, which will typically require, at most, 5,000 person-years of exposure per age group.

5 Note that in all these exercises, we assume away the possibility of individual heterogeneity, which is – perhaps to the field’s detriment – consistent with much of the empirical literature on mortality trends and patterns.

6 With annual data, this is typically the mid-year population. With five-year data (e.g., deaths in 2000–2004), this would typically be the mid-period population multiplied by five or the sum of the annual mid-year populations within the five-year time window.
